# The Effects of Two Different Ankle-Foot Orthoses on Gait of Patients with Acute Hemiparetic Cerebrovascular Accident

**DOI:** 10.1155/2014/301469

**Published:** 2014-09-09

**Authors:** Noel Rao, Jason Wening, Daniel Hasso, Gnanapradeep Gnanapragasam, Priyan Perera, Padma Srigiriraju, Alexander S. Aruin

**Affiliations:** ^1^Marianjoy Rehabilitation Hospital, 26W171 Roosevelt Road, Wheaton, IL 60187, USA; ^2^Scheck & Siress, 1551 Bond Street, Naperville, IL 60563, USA; ^3^Department of Physical Therapy (MC 898), University of Illinois at Chicago, 1919 West Taylor Street, Chicago, IL 60612, USA

## Abstract

*Objective*. To compare the effects of two types of ankle-foot orthoses on gait of patients with cerebrovascular accident (CVA) and to evaluate their preference in using each AFO type. *Design*. Thirty individuals with acute hemiparetic CVA were tested without an AFO, with an off-the-shelf carbon AFO (C-AFO), and with a custom plastic AFO (P-AFO) in random order at the time of initial orthotic fitting. Gait velocity, cadence, stride length, and step length were collected using an electronic walkway and the subjects were surveyed about their perceptions of each device. *Results*. Subjects walked significantly faster, with a higher cadence, longer stride, and step lengths, when using either the P-AFO or the C-AFO as compared to no AFO (*P* < 0.05). No significant difference was observed between gait parameters of the two AFOs. However, the subjects demonstrated a statistically significant preference of using P-AFO in relation to their balance, confidence, and sense of safety during ambulation (*P* < 0.05). Moreover, if they had a choice, 50.87 ± 14.7% of the participants preferred the P-AFO and 23.56 ± 9.70% preferred the C-AFO. *Conclusions*. AFO use significantly improved gait in patients with acute CVA. The majority of users preferred the P-AFO over the Cf-AFO especially when asked about balance and sense of safety.

## 1. Introduction

Cerebrovascular accident (CVA) is the leading cause of serious, long-term disability among adults. Each year in the United States approximately 795,000 people sustain a new or recurrent CVA [1] and nearly half survive with some level of neurological impairment and disability [2]. CVA often results in dysfunction of one side of the body (hemiparesis) leading to gait impairment and increased probability for falls [3, 4].

Restoration of ambulation and ability to move around in the community is considered a high priority for individuals after CVA [5]. An ankle-foot orthosis is commonly prescribed to assist a patient with hemiparetic CVA to return to ambulation. AFOs can prevent foot drop, control the ankle in the coronal and sagittal planes during standing and walking, and improve the stability of the knee joint during ambulation [6]. Several studies have concluded that plastic AFOs (P-AFOs) have a positive influence on the walking velocity and other gait parameters of individuals with hemiparetic CVA [7–11]. Recently carbon AFOs (C-AFOs) have been introduced to the marketplace by several manufacturers. The manufacturers of the C-AFOs claim that these devices are appropriate for hemiparetic patients who meet criteria that include minimal equinus contracture of the ankle, minimal coronal plane deformity of the ankle, minimal fluctuating edema, and no or low spasticity. However, the literature on the use of carbon AFOs to improve ambulation of individuals with CVA is insufficient. Moreover, to the best of our knowledge, no information exists on the patients' preference in using carbon AFOs.

There is also a body of literature on the importance of using a custom-made plastic AFO [12, 13]. Thus, it was reported that when a patient with posterior tibial tendon dysfunction was provided with an off-the-shelf ankle-foot orthosis (AFO), a custom solid AFO, and a custom articulated AFO, she selected a custom articulated AFO [14]. However, it is not known whether the patients with CVA would prefer using the prefabricated carbon AFOs when they have a choice to select the type of AFO. Given that the cost of orthoses varies considerably and that choosing an effective orthosis that is affordable to the patient is largely a trial-and-error process [14], it is important to obtain information on the patients' preference when prescribing an AFO.

It was reported that 35.3% of the study subjects who used an AFO indicated that they walked more confidently [15] and that patients reported a 70% increase in self-confidence [13] while using an AFO. These reports suggest a feeling of confidence may be more important to persons with hemiparesis than speed and distance; however, little information exists on the subjective preference in selecting the type of AFO by individuals with stroke.

While the literature provides important information on the beneficial effect of the AFOs, there is a need for more data describing the impact of AFOs on gait of subjects in the acute phase of rehabilitation after CVA. Moreover, there is not enough data on the use of the prefabricated carbon AFOs in gait of individuals with acute stroke. Thus, the aims of the study were (1) to investigate the effect of walking using the prefabricated carbon AFO in comparison with the custom polymer AFO and no AFO and (2) to obtain data on the users' preference in using either the prefabricated carbon AFO or the custom plastic AFO.

We hypothesized that individuals with acute CVA will improve their gait while provided with an AFO and that the improvement will be similar whether they use the prefabricated carbon AFO or the custom plastic AFO. We also hypothesized that when the subjects have a choice, they will prefer using the custom-made plastic AFO.

## 2. Methods

### 2.1. Subjects

Subjects in the acute phase of rehabilitation after hemiparetic CVA were referred by a physician during their visit to the orthotic clinic of a free-standing rehabilitation hospital. The inclusion criteria were as follows: CVA less than 12 weeks after onset, Ashworth scale score of less than 2, ability to walk 10 m without an AFO, but with an appropriate assistive device (cane or walker), a need for a custom polymer AFO for ambulation (based on evaluation by the clinic team), and ability to follow instructions. The exclusion criteria were as follows: histories of other significant neurologic or orthopedic disorders, significant coronal plane deformity or plantar flexion contracture, and minimal fluctuating edema. All subjects signed an informed consent approved by the Marianjoy Rehabilitation Hospital Institutional Review Board.

**Figure 1:**
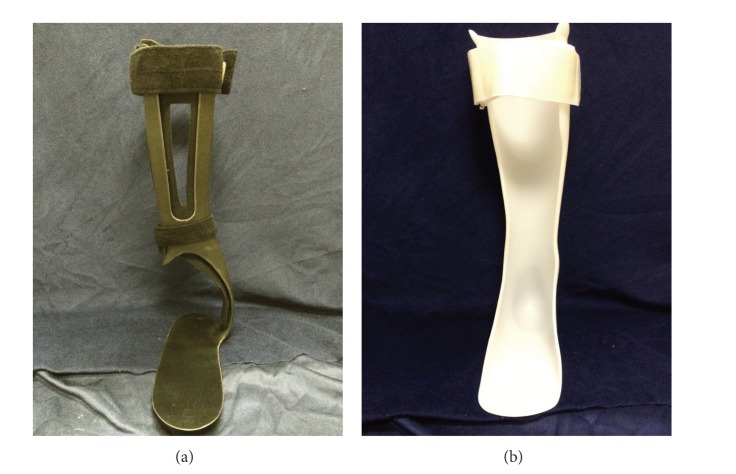
Off-the-shelf carbon AFO (a) and custom plastic AFO (b) used in the study.

Thirty individuals who satisfied inclusion/exclusion criteria were selected to participate in the study. There were 15 males and 15 females with an average age of 60.4 ± 11.3 years; 16 of the subjects presented with right and 14 with left hemiparesis. The study participants received a full clinical evaluation and assessment. Based on this assessment the clinic team determined the type of a plastic AFO (flexible, semirigid, or rigid) that was most appropriate for each subject. Subjects were molded for the appropriate AFO [16] and measured for an Ossur AFO dynamic carbon off-the-shelf AFO. Thus, based on clinical evaluation, a subject prescribed, for example, with a semirigid AFO was provided with a semirigid plastic AFO or a semirigid carbon off-the-shelf AFO. Individuals who required a different type of AFO, such as a metal AFO, were excluded from the study. Plastic AFOs were custom modified and fabricated from 3/16′′ polypropylene and included a proximal calf strap and distal ankle wrap around strap. During the course of the study subjects were provided with minimal information about the design and construction of the different AFOs. Instead, the AFOs were described as AFO A and AFO B for the subjects to prevent their impression of the device from being influenced by words such as “custom” or “carbon” (Figure 1). Subjects were tested at 21 ± 8 days after CVA. While all of the subjects were actively receiving physical and occupational therapy as prescribed by the treating physician, none of them had experience with an AFO prior to the day of fitting and testing. Subjects received clinically appropriate AFO: three subjects received a flexible AFO, twenty received a semirigid AFO, and seven subjects were provided with rigid AFOs.

### 2.2. Experimental Setup and Procedure

During the tests, the subjects were randomly provided with an off-the-shelf carbon AFO (C-AFO) and a custom plastic AFO (P-AFO). Testing included assessment of gait while walking using different AFOs and walking with no AFO (while each subject used the same own shoes during all the tests) and participation in a survey about the subject's perceptions of each AFO.

Temporal and spatial gait parameters were collected using a GAITRite electronic walkway (CIR Systems Inc., Havertown, PA). This device has been validated to study gait of individuals with stroke [17]. The *L*-test was used to further assess the subjects' functional walking ability. The *L*-test is a variant of the Timed Up and Go (TUG) test that uses an L shaped path instead of a straight line path [18]. Two walks were performed using the GAITRite and the *L*-test for each condition: no AFO, C-AFO, and P-AFO. Prior to the tests, the subjects were allowed for a brief adaptation with the AFOs. The order of the test conditions was randomized across the subjects. Subjects used an appropriate assistive device such as a cane or walker as needed; each subject used the same assistive device during all the tests involving ambulation. Subjects were allowed close supervision by a physical therapist but no direct contact. A minimum of five-minute rest between test conditions was provided to minimize the effect of fatigue.

All the study participants completed the Subject Perception of Functional Benefit Survey about their impression of the device that was just used (with assigned values of 0 being the most positive response and 4 the most negative). This survey was conducted after the use of each AFO type. In addition, at the end of the study, subjects completed the Subject AFO Preference Survey to determine if subjects had a preference for the polymer AFO, carbon fiber AFO, both AFOs, or neither AFO (with assigned values of 0 being the most positive response and 3 the most negative). These surveys are slightly modified versions of the survey described in the literature [18].

### 2.3. Data Processing and Analysis

Walking data was processed within the GAITRite software and velocity, cadence, and stride and step length were obtained as outcome measures. All the data were subjected to Shapiro-Wilk test for normality. One-way repeated measures ANOVA was performed with factor AFO (3 levels: no AFO, C-AFO, and P-AFO) separately for velocity, cadence, and stride length. Split-plot ANOVA was performed with factors AFO (3 levels: no AFO, C-AFO, and P-AFO) and side (involved and uninvolved) to analyze the differences in step length. Pairwise comparisons were used for further analyses of significant effects. Mann-Whitney test comparing survey responses for the polymer AFO and the carbon AFO was performed to test for statistical difference in subject perceptions of the functional benefit of the two different devices. For all tests, statistical significance was set at *P* < 0.05. Statistical analysis was performed in SPSS 17 for Windows 7 (SPSS Inc., Chicago, USA).

## 3. Results

### 3.1. Gait Velocity

**Figure 2:**
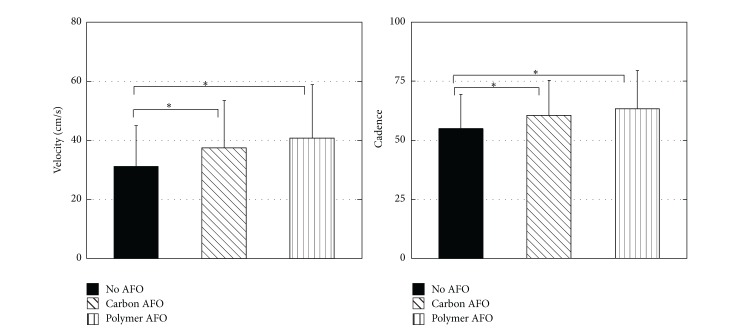
Gait velocity and cadence measured while walking without an AFO and with a carbon AFO or plastic AFO. ∗ shows statistical significance (*P* < 0.001).

With no AFO, the patients' gait velocity was 30.50 ± 13.58 cm/sec. When they were provided with the off-the-shelf carbon AFO (C-AFO), gait velocity increased to 36.38 ± 16.06 cm/sec. When a custom plastic AFO was used their gait velocity increased further reaching 39.21 ± 18.09 cm/sec. The difference in gait velocity was statistically significant (*F*_2,58_ = 20.86, *P* < 0.0001). Further analysis revealed statistically significant difference between no AFO condition and C-AFO and P-AFO (*P* = 0.001 and *P* < 0.0001, resp.). While the subjects were able to walk faster while being provided with P-AFO as compared to C-AFO, the difference was not statistically significant (*P* = 0.11) (Figure 2).

### 3.2. Cadence

While walking without AFO, the subjects' cadence was 54.93 ± 13.95 steps/min. Cadence increased while using the AFOs. Thus, it reached 60.20 ± 14.72 and 62.76 ± 15.81 steps per minute when the subjects were provided with C-AFO and P-AFO, respectively. The difference between conditions was statistically significant (*F*_2,58_ = 21.90, *P* < 0.0001). Pairwise comparison revealed that no AFO condition was statistically significant compared to C-AFO and P-AFO conditions (*P* = 0.001, *P* < 0.0001, resp.). However the difference between C-AFO and P-AFO was not significant (*P* = 0.099) (Figure 2).

### 3.3. Stride Length

Patients walking without AFOs showed stride length of 64.98 ± 16.86 cm. When they were provided with the off-the-shelf carbon AFO (C-AFO), stride length increased to 70.35 ± 18.83 cm. When a custom polymer AFO was used, the stride length increased further reaching 72.35 ± 20.11 cm. The difference in stride length was statistically significant (*F*_2,58_ = 10.23, *P* < 0.0001). Further analysis revealed statistically significant difference between no AFO condition and C-AFO and P-AFO (*P* = 0.006 and *P* < 0.01, resp.). The difference in stride length between walking while being provided with P-AFO and C-AFO was not statistically significant (*P* = 0.79).

### 3.4. Step Length

**Figure 3:**
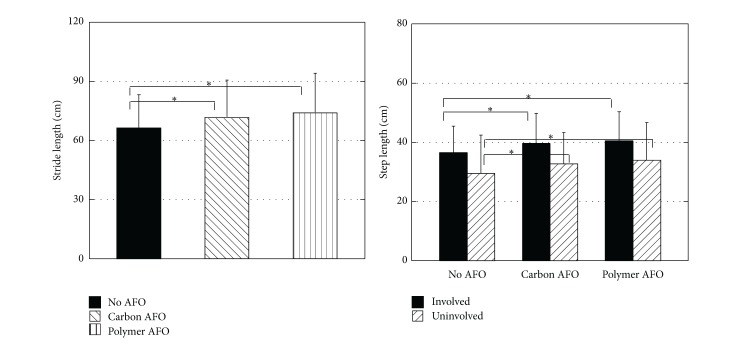
Stride length and step length recorded during walking without an AFO and with a carbon AFO or plastic AFO. ∗ shows statistical significance (*P* < 0.001).

Step length in conditions with no AFO was 36.99 ± 8.65 cm and 27.63 ± 14.01 cm for the involved and uninvolved lower extremities, respectively. When the off-the-shelf carbon AFO (C-AFO) was provided, step length increased to 39.66 ± 9.97 cm and 31.00 ± 11.66 cm on the involved and uninvolved side, respectively. Using the custom P-AFO resulted in the increase of the step length on the involved side to 40.21 ± 9.69 and on the uninvolved side to 31.92 ± 14.09. The difference in step length while provided with AFOs was statistically significant (*F*_2,116_ = 12.97, *P* < 0.0001). The interaction between the AFOs and the involved or uninvolved side was however not statistically significant (*F*_2,116_ = 0.24, *P* = 0.79). Further pairwise comparison of the use of AFOs revealed statistically significant difference between no AFO condition and C-AFO and P-AFO (*P* = 0.001 and *P* < 0.0001, resp.). The difference in step length between walking while being provided with P-AFO and C-AFO was not statistically significant (*P* = 0.99) (Figure 3).

### 3.5. *L*-Test

While walking without AFO, the time needed to cross 10 m distance by the subjects was 1.45 ± 0.71 min. This time decreased to 1.25 ± 0.79 min and to 1.33 ± 0.66 min when the subjects were provided with C-AFO and P-AFO, respectively (*L*-test data was collected for 26 subjects). The difference between conditions, however, was not statistically significant (*F*_2,52_ = 6.35, *P* = 0.14). Pairwise comparison revealed that no AFO condition was statistically significant compared to C-AFO (*P* = 0.004). At the same time, the difference between no AFO and P-AFO and between C-AFO and P-AFO was not statistically significant (*P* = 0.1417, *P* = 0.46, resp.).

### 3.6. Users' Perceptions regarding the AFO Type

The analysis of the outcome of the Perception of Functional Benefit Survey about the device that was just used revealed that in general the study participants preferred P-AFO when compared to the C-AFO (data for 29 subjects). Thus, the users demonstrated statistically significant preference of using P-AFO while answering question 2 (*U* = 280.5, *P* = 0.023), question 5 (*U* = 252, *P* = 0.006), question 6 (*U* = 271, *P* = 0.014), question 7 (*U* = 250, *P* = 0.005), and question 8 (*U* = 262.5, *P* = 0.009). Moreover, the differences between the two types of AFO were just under the level of statistical significance for question 1 (*U* = 301, *P* = 0.053) and question 4 (*U* = 301.5, *P* = 0.051) (Table 1).

The analysis of the outcome of the Subject AFO Preference Survey revealed that the study participants preferred the P-AFO to the C-AFO (Table 2). This was especially true when they were asked about balance and sense of safety during ambulation. On average, 50.87 ± 14.7% of the study participants preferred P-AFO and 23.56 ± 9.70% of the subjects preferred C-AFO. It is also important to mention that 18.1 ± 8.3% preferred using both types of AFOs and only 3.1 ± 7.90% of the study participants preferred not using either type of AFO.

## 4. Discussion

It is documented in the prior literature that individuals after CVA walk slower than healthy individuals [19]. It was also reported that the ability of individuals with stroke to ambulate is improved as a result of wearing an ankle-foot orthosis [20]. Moreover, there is a plethora of evidence on the beneficial effect of AFOs in improving of functional mobility and quality of gait as well as decreasing the likelihood of falls in individuals suffering from a CVA [21, 22]. Thus, individuals with CVA provided with an AFO improved gait parameters such as cadence, stride length, and gait velocity [18, 23, 24]. Moreover, improvement in gait velocity that is believed to reflect progress in mobility, is often used as a measure of recovery after a CVA [20, 25, 26], and is considered an important goal of rehabilitation.

At the same time, the literature on the use of different types of AFOs (especially recently introduced carbon AFOs) to improve ambulation of individuals with CVA is limited. As such, the goals of the study were to investigate the effect of walking using the prefabricated carbon AFO in comparison with the custom polymer AFO and no AFO and to investigate the users' preference in using either the prefabricated carbon AFO or the custom plastic AFO.

The results of the current study demonstrated that individuals with acute CVA increased their gait velocity when using either C-AFO or P-AFO, and, as a result, the gap between “normal” and “hemiparetic” gait velocity decreased. These results are in line with the literature reporting a positive effect of AFOs on gait velocity of individuals with hemiparesis [9, 19, 27]. Similarly, improvements in cadence were associated with the use of an AFO; without an AFO patients walked with lower cadence as compared to healthy individuals. A similar positive effect of AFOs on cadence of individuals with hemiparesis was described in the literature [27, 28]. Since cadence is the number of steps taken in a certain time, the observed increase in the velocity of gait could be due to changes in the number of steps per minute. Nevertheless, the impact of AFOs on cadence remains inconclusive as some studies report an improvement in this variable with the AFO use and others report no significant improvement [22]. Thus, there is a need for more studies focused on the investigation of the role of different types of AFOs on gait velocity and cadence. An increase in the average stride and step length with either type of AFO was evident in the current study and has been also reported in the past literature [27, 28].

While in general there is a consensus in the literature that gait velocity is a powerful indicator of function and prognosis after CVA [26] there is an opinion that gait velocity itself may not be wholly representative of functional mobility and meaningful improvement in performance [29]. To address this possible concern, we added the *L*-test assessment (that includes rising from a chair, two 90-degree turns in opposite directions, a 180-degree turn, and returning to a seated position) as the task that is indicative of the types of movements that are required in household ambulation. The outcome of the *L*-test revealed that patients while using AFOs of either type showed better performance compared to walking with no AFO. Moreover, clinically the patients showed improvement as the time needed for the completion of the test was shorter when they were provided with either type of AFO.

The study participants were positive about the use of AFOs as the majority found it comfortable and felt it improved their walking, particularly the functional aspects such as safety and confidence. This finding is in line with the literature reporting that 96% of users felt they walked better with the AFO and found it comfortable [18]. Moreover, the majority of the subjects gave a preference to the custom-made plastic AFOs: the subjects perception was about 2 : 1 in favor of the custom-made plastic AFO. This could be because each plastic AFO was specifically molded and fitted for each subject. Nevertheless, further study is needed to examine the longer-term effects and the cost-effectiveness of prescribing a custom-made or off-the-shelf AFO for people with CVA.

## 5. Conclusion

Both types of AFO significantly improved gait velocity, cadence, step length, and stride length in patients with acute CVA. The majority of users preferred the custom-made plastic AFO over the prefabricated carbon AFO. This outcome should be taken into consideration while prescribing AFOs. Further study is needed to examine the longer-term effects and the cost-effectiveness of prescribing different types of AFO for people with CVA.

## Figures and Tables

**Table 1:** Subjects perception of Functional Benefit Survey.

	With the AFO I just used…	Response					Significance
(1)	Lifting my toes is…	Much easier	A little easier	No different	A little harder	Much harder	*P* = 0.053
(2)	Swinging my leg forward is…	Much easier	A little easier	No different	A little harder	Much harder	**P** ** = 0.023**
(3)	Taking weight through my foot is…	Much easier	A little easier	No different	A little harder	Much harder	*P* = 0.105
(4)	My walking speed…	Much faster	A little faster	Not changed	A little slower	Much slower	*P* = 0.051
(5)	My balance is…	Much better	A little better	No different	A little worse	Much worse	**P** ** = 0.006**
(6)	My confidence is…	Much higher	A little higher	Not changed	A little less	Much less	**P** ** = 0.014**
(7)	My sense of safety is…	Much higher	A little higher	Not changed	A little less	Much less	**P** ** = 0.005**
(8)	Walking is…	Much easier	A little easier	No different	A little harder	Much harder	**P** ** = 0.009**

**Table 2:** Subjects perceptions regarding the AFO type, *N* = 29.

		Plastic AFO	Carbon AFO	Both	None
(1)	Lifting my toes is easier with…	8 (27%)	5 (17%)	14 (48%)	1 (3%)
(2)	Swinging my leg forward is easier with	17 (58.6%)	8 (27%)	4 (13.8%)	0 (0%)
(3)	Taking weight through my foot is easier with…	16 (55%)	8 (27%)	5 (17%)	0 (0%)
(4)	I walk faster with…	18 (62%)	5 (17%)	6 (21%)	0 (0%)
(5)	My balance is better with…	18 (62%)	5 (17%)	6 (21%)	0 (0%)
(6)	My sense of safety is higher with…	17 (58.6%)	2 (7%)	10 (34%)	0 (0%)
(7)	I like the fit and comfort of…	15 (52%)	11 (38%)	2 (7%)	1 (3%)
(8)	I like the appearance of…	17 (58.6%)	9 (31%)	3 (10%)	0 (0%)
(9)	I would rather use…to assist my walking	7 (24%)	9 (31%)	6 (21%)	7 (24%)
